# MRGCN: cancer subtyping with multi-reconstruction graph convolutional network using full and partial multi-omics dataset

**DOI:** 10.1093/bioinformatics/btad353

**Published:** 2023-05-31

**Authors:** Bo Yang, Yan Yang, Meng Wang, Xueping Su

**Affiliations:** The Shaanxi Key Laboratory of Clothing Intelligence, School of Computer Science, Xi’an Polytechnic University, Xi’an 710048, China; Donnelly Centre for Cellular and Biomolecular Research, University of Toronto, Toronto, ON M5S 3E1, Canada; The Shaanxi Key Laboratory of Clothing Intelligence, School of Computer Science, Xi’an Polytechnic University, Xi’an 710048, China; The Shaanxi Key Laboratory of Clothing Intelligence, School of Computer Science, Xi’an Polytechnic University, Xi’an 710048, China; School of Electronics and Information, Xi’an Polytechnic University, Xi’an 710048, China

## Abstract

**Motivation:**

Cancer is a molecular complex and heterogeneous disease. Each type of cancer is usually composed of several subtypes with different treatment responses and clinical outcomes. Therefore, subtyping is a crucial step in cancer diagnosis and therapy. The rapid advances in high-throughput sequencing technologies provide an increasing amount of multi-omics data, which benefits our understanding of cancer genetic architecture, and yet poses new challenges in multi-omics data integration.

**Results:**

We propose a graph convolutional network model, called MRGCN for multi-omics data integrative representation. MRGCN simultaneously encodes and reconstructs multiple omics expression and similarity relationships into a shared latent embedding space. In addition, MRGCN adopts an indicator matrix to denote the situation of missing values in partial omics, so that the full and partial multi-omics processing procedures are combined in a unified framework. Experimental results on 11 multi-omics datasets show that cancer subtypes obtained by MRGCN with superior enriched clinical parameters and log-rank test *P*-values in survival analysis over many typical integrative methods.

**Availability and implementation:**

https://github.com/Polytech-bioinf/MRGCN.git https://figshare.com/articles/software/MRGCN/23058503.

## 1 Introduction

Cancer is a large family of diseases that can originate in almost any organ or tissue of the human body when abnormal cells grow uncontrollably, that is, beyond the usual boundaries, invade adjacent areas of the body, and/or spread to other organs (Hejmadi 2014). The traditional prediction of cancer is greatly influenced by morphological evaluation of tumor, whereas some tumors with similar histopathological appearance present remarkably different clinical manifestations, courses, and even outcome of therapy. The heterogeneity of cancer becomes the major resistance of the development of effective therapies (Pasha and Turner 2021). In some instances, the heterogeneity is traceable in the fact that morphologically similar tumors have several subtypes with distinct pathogeneses and clinical features. Cancer subtyping could effectively deal with interpatient heterogeneity by stratifying patients into distinct groups in terms of risk factors and clinical prognosis. Consequently appropriate cancer subtyping could induce target specific therapies and help in providing more efficient treatment and minimizing toxicity on the patients.

The progress in high-throughput sequencing technologies has provided the collection of various types of omics data with unprecedented details. Some large national and international consortia, such as The Cancer Genome Atlas (TCGA), have collected thousands of biological tumor samples data from multiple molecular events. Integrating and analyzing these multi-omics data representing information from different molecular processes could improve holistic view of understanding of the complex biology. Specifically, cancer is accumulation of mutations and epimutations (Lynch *et al.* 2015, Belizario and Loggulo 2019) and its heterogeneity results from genetic, transcriptomic, epigenetic, and phenotypic changes. Thus cancer subtyping using multi-omics data has been the crux of cancer diagnosis, prognosis and treatment.

A large number of multi-omics data integration methods have been proposed over the years (Subramanian *et al.* 2020, Duan *et al.* 2021). Most existing schemes adopt unsupervised strategy, since supervised methods are based on annotating samples, which requires time consuming and laborious clinical follow-up, e.g. MoGCN (Li *et al.* 2022) adopts supervised GCN to achieve patient classification. In addition, supervised methodology assigns individual cancer samples to already-defined subtypes, but the subtypes definition is still an open problem (Popovici *et al.* 2017). Clustering-based methods are not required to know the class labels in training, but just via calculating the similarity in samples to obtain subgroups division of patients. Early attempts of clustering-based integration algorithms involve feature concatenation-based strategies and ensemble-based strategies (Tini *et al.* 2019). The feature concatenation-based algorithms integrate data attribute from different omics using the form of series connection directly and run conventional clustering method, e.g. *K-*means (Hartigan and Wong 1979) and spectral clustering (Von Luxburg 2007) on the integrated data. A typical way of feature concatenation-based algorithms is LRAcluster (Wu and Lai 2015). The ensemble-based algorithms fuse the prediction results from different clustering models trained on each type of omics data individually, e.g. CC (Monti 2003) and PINS (Nguyen *et al.* 2017). However, these algorithms ignore the correlations among different omics data types. Recently, many integration algorithms try to construct a holistic representation learning model for exploiting the interactions across different omics data types and have gradually become mainstream. For example, MCCA (Witten and Tibshirani 2009) adopts sparse canonical correlation analysis to find highly correlated omics data. iCluster (Shen *et al.* 2009) develops a joint Gaussian latent variable model to express multi-omics data as sparse linear codes on an inherent low dimensional representation. iClusterBayes (Mo *et al.* 2018) tries to find a few latent variables via Bayesian variable selection and describes the inherent structure in multiple omics data. SNF (Wang *et al.* 2014) constructs neighborhood graph of samples for each omics data individually and then uses message passing theory to fuse these graphs into a uniform similarity network. SNFCC (Xu *et al.* 2017) combines SNF and CC algorithms to predict the cancer subtypes. NEMO (Rappoport and Shamir 2019) constructs one similarity matrix for each omics data using radial basis function kernel and averages all similarity matrices to achieve integration. MSNE (Xu *et al.* 2021) utilizes random walk on multiple networks to integrate similarity of samples and then projects the samples into a low-dimensional space.

Clustering aims at dividing a group of unlabeled data into several disjoint groups, such that the data in the same group with high correlation to each other (Xia *et al.* 2022). Hence, preserving the similarity relationship of samples plays a critical role in the clustering task. Graph Convolutional Network (GCN) recently has been shown very effective in clustering, since it calculates the embedding representation by incorporating preservation of graph architecture reflecting the similarity relationships. The representative GCN models include graph auto-encoder (Kipf *et al.* 2016), adversarial regularized graph auto-encoder (Pan *et al.* 2018), deep attentional embedded graph clustering approach (Wang *et al.* 2019), etc. The decoder parts in abovementioned models reconstruct the graph structure by using the inner product of the leaned embedding representation. This strategy is merely applicable to single view scenario, as there is only one graph structure need to be reconstructed. When confronted with the multi-omics data, i.e. multi-view learning problems, there are several graph structures need to be reconstructed, the inner product strategy would be invalidated, since inner product of the consistent representation of multi-view data would generate one and the only one reconstruction graph result. In addition, in multi-omics analyses, there is a common phenomenon that some samples only have measurements for a subset of the omics (Rappoport and Shamir 2019). The sample missing some omics data is called partial sample, and the multi-omics dataset including the partial sample is called partial dataset. The traditional GCN can only deal with full datasets, i.e. data from all omics were measured for each patient, but cannot handle the partial datasets without imputation.

Inspired by above insight analysis and the fact that GCN is able to capture the nonlinear inherent representation, meanwhile preserves similarity relationship but cannot deal with partial multi-omics datasets, we propose Multi-Reconstruction Graph Convolutional Network (MRGCN) to identify cancer subtypes. First, MRGCN constructs one graph for each omics data using neighborhood relationships and encodes each omics data to obtain individual embedding representation. Second, MRGCN builds an indicator matrix to express the data missing situation and integrates each individual embedding into a consensus representation. Third, MRGCN decodes the consensus representation to reconstruct graph structures and node attribute simultaneously. Furthermore, MRGCN adopts self-supervised learning mechanism to enhance the discriminability of consensus representation. Finally, all aforementioned parts are incorporated into a joint optimization problem and solved by the deep learning framework. The cancer subtyping is carried out via spectral clustering based on the obtained consensus representation.

To our best knowledge, MRGCN is the first attempt at using GCN to simultaneously reconstruct graph structures and node attribute obtaining the latent representation in both full and partial multi-omics data. Extensive computational experiments on 11 datasets demonstrate the superiority of MRGCN in cancer subtyping capability over corresponding solutions to multi-omics data integration.

## 2 Materials and methods

MRGCN includes four modules, i.e. individual encoder, consensus representation, node attribute reconstruction, and graph structures reconstruction. Cancer subtyping is carried out on the consensus representation via spectral clustering algorithm. Each module and step will be detailed in the following sections.

### 2.1 Notation

Let $X=\left\{X^{\left(1\right)},X^{\left(2\right)},\ldots ,X^{\left(V\right)}\right\}$ denote a multi-omics dataset, where *V* is the number of omics. $X^{\left(v\right)}={\left[x_{1}^{\left(v\right)},x_{2}^{\left(v\right)},\ldots ,x_{N_{v}}^{\left(v\right)}\right]}^{T}\in R^{N_{v}\times D_{v}}$ is a collection of $N_{v}$ data samples with dimension $D_{v}$ in *v*th omics measurements, where v=1,2,…,V. $A=\left\{A^{\left(1\right)},A^{\left(2\right)},\ldots ,A^{\left(V\right)}\right\}$ is the corresponding graph structure matrix set, where $A^{\left(v\right)}\in R^{N_{v}\times N_{v}}$. The consensus representation is $H={\left[h_{1},h_{2},\ldots ,h_{N}\right]}^{T}\in R^{N\times d}$, where *d* is the ultimate dimension of consensus embedding space. *N*, $N\geq N_{v}$ is the sample size of intact data and $||\cdot |{|}_{F}^{2}$ is the Frobenius norm.

### 2.2 The framework of MRGCN

**Figure 1:**
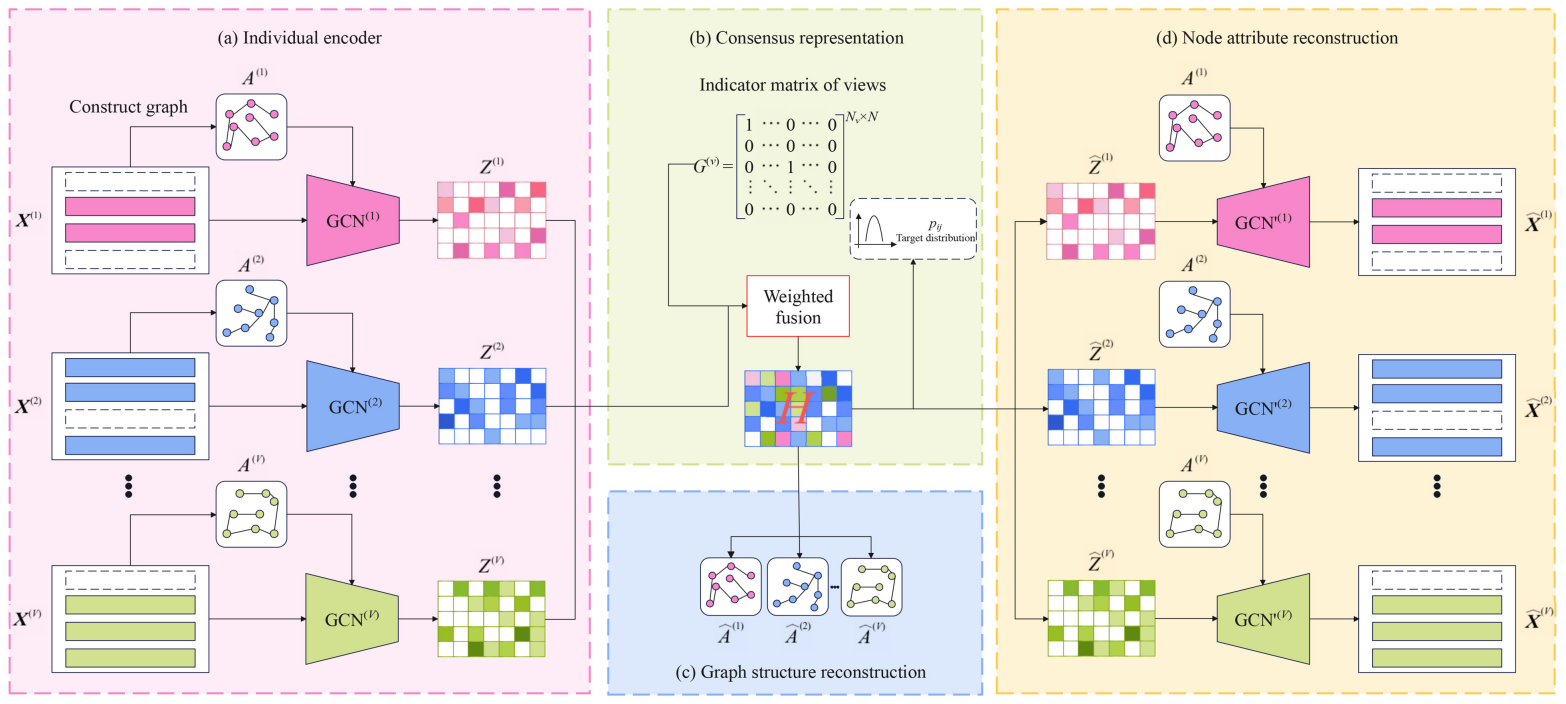
The framework of MRGCN model. (a) Individual encoder is used to learn embedding from node attribute and graph relationship of each omics data. (b) Consensus representation is used to integrate each embedding from each omics data into a shared space and meanwhile to handle the partial sample missing problem. (c) Graph structure reconstruction is used to reconstruct the similarity relationship of each omics data. (d) Node attribute reconstruction is used to reconstruct omics expression.

As shown in Fig. 1, MRGCN contains four principal modules. First, each omics data $X^{\left(v\right)}$ and the corresponding graph structures $A^{\left(v\right)}$ are encoded into $Z^{\left(v\right)}$ via the individual encoder module. Then, $Z^{\left(v\right)}$ is fed into consensus representation module and obtain ***H***. Finally, the node attribute reconstruction module and graph structure reconstruction module achieve multi-reconstruction conducted on ***H***.

#### 2.2.1 Individual encoder

One similarity graph is constructed for each omics data, as follows: where $\mathrm{Nei}\left(x_{j}^{\left(v\right)}\right)$ denotes the neighbor set of $x_{j}$ in *v*th omics measurements. The individual encoder of each omics is a nonlinear function $f\left(A^{\left(v\right)},X^{\left(v\right)}|W^{\left(v\right)}\right)\to Z^{\left(v\right)}$, that maps *v*th omics data attribute $X^{\left(v\right)}$ and corresponding similarity graph structure $A^{\left(v\right)}$ into the individual embedding $Z^{\left(v\right)}$, where $W^{\left(v\right)}$ is parameters in individual encoder. The outputs of *m*th encoder layer are computed as follows: where m=1,2,…,M and *M* is the layer number of encoder. $A^{{\left(v\right)}^{\prime }}=A^{\left(v\right)}+I$ and ***I*** is the identity matrix. $D_{ii}^{\left(v\right)}=\sum_{j}A_{ij}^{{\left(v\right)}^{\prime }}$ and $W_{m}^{\left(v\right)}$ is the encoder model parameters of *m*th layer need to be determined by training. φ is the nonlinear activation function, which is set as tanh function in MRGCN. With respect to $Z_{m}^{\left(v\right)}$, when *m *=* *1, let $Z_{0}^{\left(v\right)}=X^{\left(v\right)}$, which is the original *v*th omics data, and when *m *=* M*, let $Z^{\left(v\right)}=Z_{M}^{\left(v\right)}$, which is the individual embedding of *v*th omics data. It is noteworthy that the individual embedding of each omics data should has the same feature dimension, that is, the numbers of columns for each $Z^{\left(v\right)}={\left[z_{1}^{\left(v\right)},z_{2}^{\left(v\right)},\ldots ,z_{N}^{\left(v\right)}\right]}^{T}$ are all equal to *d*.


(1) $$\begin{array}{c} A_{ij}^{\left(v\right)}=\{\begin{array}{c} 1,\;\text{if}\;x_{i}^{\left(v\right)}\in \mathrm{Nei}\left(x_{j}^{\left(v\right)}\right)\;\text{or}\;x_{j}^{\left(v\right)}\in \mathrm{Nei}\left(x_{i}^{\left(v\right)}\right)\\ 0,\;\text{otherwise}\; \end{array} \end{array}$$

(2) $$\begin{array}{c} Z_{m}^{\left(v\right)}=\varphi \left(D^{{\left(v\right)}^{-\frac{1}{2}}}A^{{\left(v\right)}^{\prime }}D^{{\left(v\right)}^{-\frac{1}{2}}}Z_{m-1}^{\left(v\right)}W_{m}^{\left(v\right)}\right) \end{array}$$

#### 2.2.2 Consensus representation

The measurements from different omics reflect different aspects of the same disease, but they also with the consensus sematic information, such as the same cluster label distribution or consensus representation (Hao *et al.*, 2021; Wen *et al.* 2021). However, in the clinical domain, patients who need to be diagnosed might miss some omics measurements, hence we design an indicator matrix ***G*** to demonstrate the data missing situation, as follows:


(3) $$\begin{array}{c} G_{ij}^{\left(v\right)}=\{\begin{array}{c} 1,\;\text{if}\;i-\text{th sample in}\;X^{\left(v\right)}\;\text{is the}\;j-\text{th sample in the intact data}\;\\ 0,\;\text{otherwise}\; \end{array} \end{array}$$

The consensus representation ***H*** shared by all omics can be established by a weighted fusion manner:


(4) $$\begin{array}{c} h_{j}=\frac{\sum_{v=1}^{V}\sum_{i=1}^{N_{v}}G_{ij}^{\left(v\right)}z_{i}^{\left(v\right)}}{\sum_{v=1}^{V}\sum_{i=1}^{N_{v}}G_{ij}^{\left(v\right)}}. \end{array}$$

#### 2.2.3 Node attribute reconstruction

The original multi-omics data through encoder layers and fusion operation is represented as ***H***, which preserves the main information in multi-omics measurements and the similarity relationships in patients. Then in decoding, the node attribute and graph structures need to be reconstructed simultaneously based on ***H***. In attribute reconstruction, decoder attempts to be a reverse of encoder, hence the layer number of decoder is also set to be ***M***. The attribute reconstruction is calculated as follows:


(5) $$\begin{array}{c} {\hat{Z}}_{M}^{\left(v\right)}=\varphi \left(H{\hat{W}}_{M}^{\left(v\right)}\right), \end{array}$$

(6) $$\begin{array}{c} {\hat{Z}}_{m-1}^{\left(v\right)}=\varphi \left(D^{{\left(v\right)}^{-\frac{1}{2}}}A^{{\left(v\right)}^{\prime }}D^{{\left(v\right)}^{-\frac{1}{2}}}{\hat{Z}}_{m}^{\left(v\right)}{\hat{W}}_{m-1}^{\left(v\right)}\right). \end{array}$$

The loss of node attribute reconstruction is defined as: where ${\hat{X}}^{\left(v\right)}$ is the output of decoder last layer, i.e. ${\hat{Z}}_{0}^{\left(v\right)}$. ${\hat{W}}_{m}^{\left(v\right)}$ are the decoder model parameters.


(7) $$\begin{array}{c} L_{\mathrm{nar}}=\sum_{v=1}^{V}{\left|\left|X^{\left(v\right)}-{\hat{X}}^{\left(v\right)}\right|\right|}_{F}^{2} \end{array}$$

#### 2.2.4 Graph structure reconstruction

The reconstructed similarity graph ${\hat{A}}^{\left(v\right)}$ of each omics data can be presented as follows: where ${\tilde{W}}^{\left(v\right)}$ is the model parameters determined by training for reconstructing the graph ${\hat{A}}^{\left(v\right)}$. Correspondingly, the loss of graph structure reconstruction can be written as:


(8) $$\begin{array}{c} {\hat{A}}^{\left(v\right)}=\varphi \left(H{\tilde{W}}^{\left(v\right)}H^{T}\right) \end{array}$$

(9) $$\begin{array}{c} L_{\mathrm{gsr}}=\sum_{v=1}^{V}{\left|\left|A^{\left(v\right)}-{\hat{A}}^{\left(v\right)}\right|\right|}_{F}^{2}\cdot \end{array}$$

### 2.3 Clustering for cancer subtyping

The cancer subtyping is achieved by spectral clustering method, and the clustering results in turn help to enhance the discriminability of consensus representation via self-supervised learning mechanism.

#### 2.3.1 Self-supervised learning mechanism

Self-supervised is a type of unsupervised learning methodology, in which the model parameters are trained with supervisory information generated from the data itself (Liu *et al.* 2022). Specifically, during training phase, some pseudo labels are generated based on attributes of data and clustering algorithm. Then the model is trained via supervised learning manner by using these pseudo labels as supervised information. Finally, update pseudo labels and retrain the model to fine tune parameters. The loss function of self-supervised learning in MRGCN is defined as follows: where *C* is the number of clusters. KL(·||·) is Kullback–Leibler divergence that measures the distance between two distributions. ***Q*** is the distribution of the soft labels, in which $Q_{ij}$ is measured by Student’s *t*-distribution (Van der Maaten and Hinton 2008) for indicating the similarity between the consensus representation $h_{i}$ and cluster center $\mu_{j}$: $P_{ij}$ in Equation (10) is the target distribution of ***Q***, defined as:


(10) $$\begin{array}{c} L_{ss}=KL(P||Q)=\sum_{i=1}^{N}\sum_{j=1}^{C}P_{ij}\;\text{log}\;\frac{P_{ij}}{Q_{ij}} \end{array}$$

(11) $$Q_{ij}=\frac{{\left(1+{\left|\left|h_{i}-\mu_{j}\right|\right|}^{2}\right)}^{-1}}{\sum_{j^{\prime }=1}^{C}{\left(1+{\left|\left|h_{i}-\mu_{j^{\prime }}\right|\right|}^{2}\right)}^{-1}}$$

(12) $$P_{ij}=\frac{Q_{ij}^{2}\sum_{i=1}^{N}Q_{ij}}{\sum_{j^{\prime }=1}^{C}\left(Q_{ij^{\prime }}^{2}/\sum_{i=1}^{N}Q_{ij^{\prime }}\right)}.$$

Minimizing KL divergence between ***Q*** and ***P*** makes the distribution of ***Q*** denser, which is particularly beneficial to enhance the discriminative ability of consensus representation.

#### 2.3.2 The overall loss function of MRGCN

MRGCN attempts to obtain embedding results for improving the clustering performance via preserving the information in data attribute and similarity relationship among samples. Therefore, the overall loss function of MRGCN then reads: where *α* and *β* are trade-off parameters.


(13) $$\begin{array}{c} L=L_{\mathrm{nar}}+\alpha L_{\mathrm{gsr}}+\beta L_{ss}. \end{array}$$

#### 2.3.3 Spectral clustering

A similarity matrix ***S*** of consensus representation ***H*** is constructed: where *t *>* *0 is a tuning parameter. The diagonal matrix ***D*** and the Laplacian matrix ***L*** are constructed as follows:


(14) $$\begin{array}{c} S_{ij}=\{\begin{array}{c} \;\text{exp}\;\left(-{\left|\left|h_{i}-h_{j}\right|\right|}_{2}^{2}/t\right),\;\text{if}\;h_{i}\in \mathrm{Nei}\left(h_{j}\right)\;\text{or}\;h_{j}\in \mathrm{Nei}\left(h_{i}\right),\\ 0,\;\text{otherwise}\;, \end{array} \end{array}$$

(15) $$\begin{array}{c} L=I-D^{-1/2}SD^{-1/2},D_{ii}=\sum_{ij}S_{ij}. \end{array}$$

The clustering results can be determined by solving the optimization problem (Liu *et al.* 2018) as follows: where ***I*** is the identity matrix, $B=Y{\left(Y^{T}Y\right)}^{-1/2},\;Y={\left[y_{1}^{T},y_{2}^{T},\ldots ,y_{N}^{T}\right]}^{T}$. $y_{i}$ is the clustering results, i.e. $y_{i}(k)=1$ denotes that *i* th patient should related to the *k*th cancer subtype. The number of clusters is determined by the modified eigengap method(Von Luxburg 2007). That is, test each value in the range [2,15] in increments of 1, and set the number of clusters to *i*, which could reach the $\arg{\;\max}_{i}\left(\lambda_{i+1}-\lambda_{i}\right)i$, where *λ_i_* is the *i*th eigenvalue of matrix ***L***.


(16) $$\begin{array}{c} \min_{B}\text{Trace}\left(B^{T}\mathrm{LB}\right),\;\\ s.t.B^{T}B=I,\; \end{array}$$

The training process consists of two parts. The first one is the pre-training, in which self-supervised learning mechanism is omitted, and only uses $L_{\mathrm{nar}}+\alpha L_{\mathrm{gsr}}$ in training. The second part is fine-tuning, in which spectral clustering is carried out on the consensus representation results obtained from pre-training and then the complete loss function, i.e. Equation (13) is used to train the model. Furthermore, in fine-tuning, the model parameters are initialized to the results of pre-training. The back propagation algorithm with stochastic gradient descent is used in all training. The detailed procedure is summarized in Algorithm1.

Algorithm 1 The MRGCN algorithm.

**Input:** Multi-level data X.

**Output:** Consensus representation ***H*** and clustering results ***Y***.

1: Construct a graph for each omics data by Equation (1).

2: Construct matrix $G^{\left(v\right)}$ by Equation (3).

3: Pre-train using loss function $L=L_{\mathrm{nar}}+\alpha L_{\mathrm{gsr}}$.

4: Calculate clustering results by Equations (14)–(16).

5: **Repeat**.

6: Fine-tune using loss function Equation (13).

7: Calculate clustering results by Equations (14)–(16).

8: **Until** convergence.

9: **Return *H*** and ***Y***.

### 2.4 Materials

Ten cancer types data from TCGA are used for evaluation, including acute myeloid leukemia (AML), breast invasive carcinoma (BIC), colon adenocarcinoma (COAD), glioblastoma multiforme (GBM), kidney renal clear cell carcinoma (KIRC), liver hepatocellular carcinoma (LIHC), lung squamous cell carcinoma (LUSC), ovarian serous cystadenocarcinoma (OV), skin cutaneous melanoma (SKCM), and sarcoma (SARC). Three omics levels are adopted for integration, including DNA methylation, mRNA and miRNA expression. In addition, the Molecular Taxonomy of Breast Cancer International Consortium (METABRIC) breast cancer dataset (Pereira *et al.* 2016) is also used for integrating mRNA and CNV expression. All data are preprocessed following (Rappoport and Shamir 2018, 2019), and the detail of datasets is summarized in Supplementary Table S1.

## 3 Results

The proposed method MRGCN is compared with 11 integrative methods on full datasets and 3 related methods on partial datasets, respectively.

### 3.1 Full multi-omics datasets

Several computational experiments were performed to evaluate the effectiveness of cancer subtyping via multi-omics data. We compare our MRGCN to 11 methods on full multi-omics datasets, including two directly clustering methods, i.e. *K*-means and spectral clustering, which conduct clustering operation on the concatenated multi-omics data, as well as nine integrating methods, i.e. LRAcluster, CC, PINS, MCCA, iClusterBayes, SNF, SNFCC, MSNE, and NEMO. The survival analysis and enrichment analysis of clinical labels are utilized to assess the performance of subtyping (Rappoport and Shamir 2019). For survival analysis, Cox proportional hazards model (Hosmer *et al.* 1999) and *P*-value are selected to indicate statistically significant difference existence in survival profiles between different cancer subtypes. For enrichment analysis of clinical labels, a unified set of patients’ clinical information is selected for all cancers, such as gender and age at initial diagnosis, as well as four discrete clinical pathological parameters quantifying the progression of the tumor (pathologic T), cancer in lymph nodes (pathologic N), metastases (pathologic M), and total progression (pathologic stage). The number of clusters within comparison methods is set to be the same value reported in the original papers, as suggested by Rappoport and Shamir (2019), details shown in Supplementary Table S2.

Competitive methods are realized using the publicly available code. The details of hyper-parameters in codes are described in Supplementary Table S3. The silhouette value is adopted as a criterion for parameters selection for each method. In MRGCN training, the learning rate is set to 0.001. For simplicity, the parameters *α* and *β* are both set to 1. If the lowest dimension in each omics expression for given dataset is <2000, the dimension of consensus representation *d* is set to be 0.8× the lowest dimension, and otherwise set to be 1600, details shown in Supplementary Table S4. The number of nodes in each layer of MRGCN is displayed in Supplementary Tables S5 and S6. After the input features have been determined, normalized *z*-score (Cheadle *et al.* 2003) is used to achieve normalization. The training time of different methods is show in Supplementary Fig. S1. Since our method MRGCN is based on deep learning, it requires more time to train neural networks. However, compared with iClusterBayes and CC, the time consumption of MRGCN is still acceptable.

**Figure 2:**
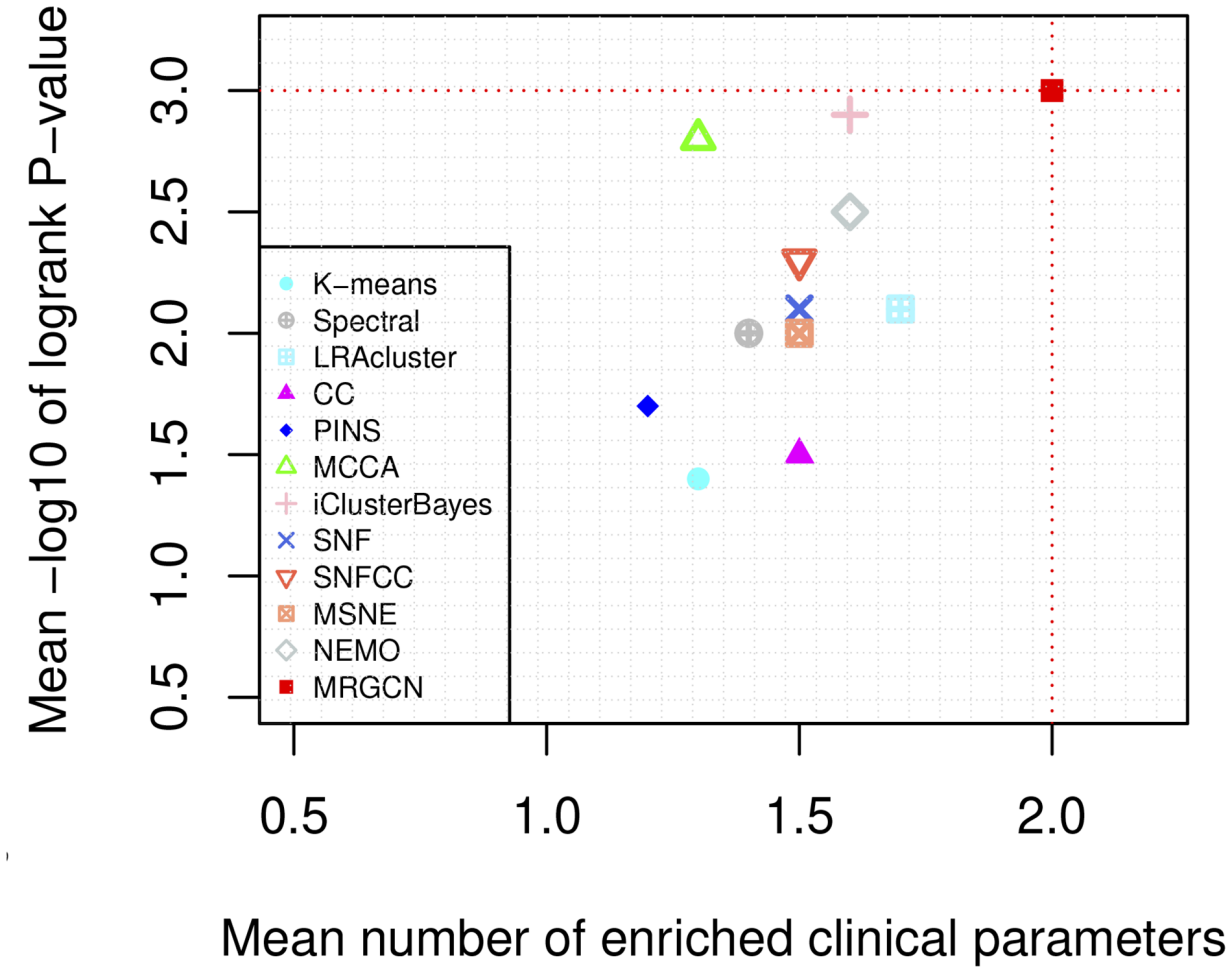
Mean performance of the different algorithms on 11 cancer datasets. *Y*-axis represents average −log10 logrank test’s *P*-values and *X*-axis represents average number of enriched clinical parameters in the clusters. The red dotted lines highlight the results of MRGCN.

Table 1, Fig. 2, and Supplementary Table S7 summarize the cancer subtyping performance of different algorithms on 10 full TCGA datasets and one METABRIC dataset. It is clearly observed that the clusters found by MRGCN with significant difference in survival for 9 of the 11 cancer datasets. The average logrank *P*-value of MRGCN reaches to 3.0. iClusterBayes is the second with 2.9. None of the methods found clusters with significantly different survival for COAD and OV datasets. MRGCN found at least one enriched clinical parameter in all datasets. LRAcluster, CC, iClusterBayes, SNF, SNFCC and MSNE are tied for second with 10. Furthermore, the average number of enriched clinical parameters of MRGCN is 2.0, while LRAcluster is the second with 1.7. These results demonstrate MRGCN could identify significant coherent and clinically relevant patient subtypes.

**Table 1.** The comparisons of clustering results from different algorithms on full TCGA and METABRIC datasets.^a^

Alg./cancer	AML	BIC	COAD	GBM	KIRC	LIHC	LUSC	OV	SKCM	SARC	METABRIC	Mean	Sig
*K*-means	1/**2.4** (5)	2/**3.5** (4)	1/0.4 (2)	2/**2.6** (5)	1/0.8 (2)	2/0.2 (2)	0/**1.5** (2)	1/0.3 (2)	2/0.9 (2)	2/1.3 (2)	0/1.2 (5)	1.3/**1.4**	9/4
Spectral	1/**2.4** (6)	1/**5.4** (3)	1/0.9 (12)	2/**2.6** (5)	3/**1.5** (3)	2/0.4 (2)	0/**2.1** (2)	1/0.8 (4)	2/**1.4** (6)	2/1.3 (2)	0/**3.5** (7)	1.4/**2.0**	9/7
LRAcluster	1/**1.8** (7)	2/**5.6** (5)	1/0.8 (10)	2/1.3 (12)	4/1.3 (11)	2/**2.4** (12)	1/1.0 (12)	1/0.4 (4)	3/**2.9** (15)	2/**2.5** (13)	0/**2.7** (7)	1.7/**2.1**	10/6
CC	1/**3.8** (3)	1/**2.8** (5)	1/0.5 (2)	2/**2.1** (7)	3/1.3 (4)	2/0.5 (2)	1/1.1 (4)	0/0.2 (3)	3/**2.5** (4)	2/1.0 (2)	1/1.0 (6)	1.5/**1.5**	10/4
PINS	1/**2.0** (4)	1/**4.1** (5)	0/0.5 (4)	1/**4.4** (2)	3/**1.7** (6)	2/0.8 (5)	0/**2.0** (2)	0/0.1 (2)	2/1.0 (15)	2/0.8 (3)	1/**1.8** (7)	1.2/**1.7**	8/6
MCCA	1/**2.1** (12)	1/**8.0** (5)	0/0.3 (2)	1/**2.9** (11)	2/**1.8** (15)	2/1.2 (15)	2/**2.3** (12)	0/0.6 (9)	2/**4.7** (2)	2/**1.5** (15)	1/**5.7** (7)	1.3/**2.8**	9/8
iClusterBayes	1/**2.0** (5)	2/**3.2** (4)	2/0.1 (2)	1/**3.1** (2)	4/**7.3** (2)	2/**3.3** (6)	0/**1.6** (5)	1/1.0 (6)	2/0.6 (2)	2/**3.7** (2)	1/**5.7** (7)	1.6/**2.9**	10/8
SNF	1/**3.2** (6)	2/**6.3** (5)	1/0.5 (3)	2/**2.6** (2)	3/**1.7** (4)	2/1.1 (5)	1/**1.5** (2)	1/0.6 (3)	1/1.1 (3)	2/**1.9** (3)	0/**2.6** (7)	1.5/**2.1**	10/7
SNFCC	1/**4.0** (4)	3/**7.5** (5)	2/0.6 (10)	2/**2.3** (9)	2/**1.5** (2)	1/1.2 (10)	1/**1.7** (2)	0/0.5 (3)	2/1.3 (4)	2/**1.7** (3)	1/**2.7** (7)	1.5/**2.3**	10/7
MSNE	1/**3.2** (5)	2/**3.8** (4)	1/0.3 (5)	1/**3.0** (2)	2/**1.5** (4)	3/1.2 (5)	1/**1.5** (2)	0/0.5 (4)	2/**2.0** (4)	2/**1.8** (3)	1/**2.7** (9)	1.5/**2.0**	10/8
NEMO	1/**1.8** (5)	2/**4.2** (4)	0/0.1 (3)	1/**3.8** (4)	4/**2.2** (12)	4/**4.2** (5)	0/**1.8** (2)	0/0.4 (3)	3/**4.0** (5)	2/**1.9** (3)	1/**3.5** (9)	1.6/**2.5**	8/9
MRGCN	1/**3.0** (10)	4/**6.7** (4)	1/0.6 (7)	2/**3.8** (8)	4/**2.4** (9)	2/**1.7** (10)	1/**1.5** (13)	1/0.8 (5)	3/**4.5** (5)	2/**3.3** (8)	1/**5.2** (9)	2.0/**3.0**	11/9

a In each cell A/B (C), A is significant clinical parameters detected. B is −log10 *P*-value for survival. C is the number of clusters. 0.05 is the threshold for significance and the bold indicates the significant results. Mean is algorithm average value. Sig is the number of datasets with significant results.

The visualization of the consensus representation from MRGCN on BIC dataset by t-SNE and UMAP is shown in Supplementary Fig. S2. From the figure, we clearly see that the proposed MRGCN has good discriminant capability, since the intra-cluster compactness and the inter-cluster separation are achieved at the same time. Supplementary Fig. S3 shows the survival curves of the patients in the subtypes identified by each method on BIC. From the figure, we can see that MRGCN has significantly lower *P*-values than other comparison methods, which indicates the MRGCN is better than the other existing methods.

**Figure 3:**
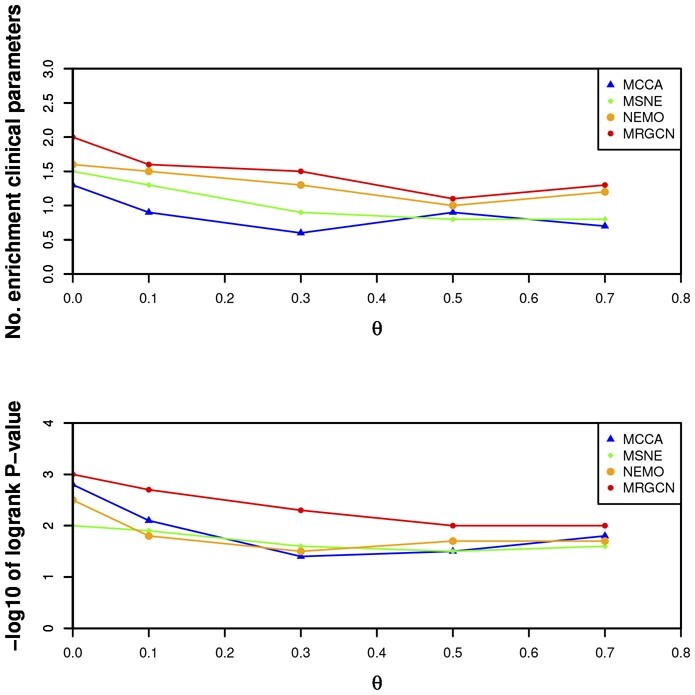
Average performance as a function of the missing fraction. The top plot shows the results of enriched clinical parameters and the bottom plot shows the results of survival analysis.

In order to compare the subtypes obtained by MRGCN and the existing subtypes, meanwhile to display the differential expression among different subtypes, we designed the experiments with following processes. First, subtyping results of PAM50 classification (Parker *et al.* 2009) on BIC dataset are selected for comparison. Second, for MRGCN on BIC dataset, we found that there are 48 features in mRNA expression related to the 50 genes of PAM50, and then delete the 48 features in mRNA expression, with the aim of eliminating direct effects of the 50 known oncogenes in multi-omics integration. Third, the processed mRNA data together with anther omics data are input to MRGCN model. Finally, the expressions of 48 mRNA are used to draw heatmap for showing the correlation of oncogenes with the obtained subtypes from MRGCN, as well as the overlap of subtypes from MRGCN and PAM50 classification. Supplementary Fig. S4 shows the heatmap results, in which samples are rearranged by subtypes from MRGCN. It could be observed that different subtypes have distinct expression patterns and there are some overlap subtypes between MRGCN and PAM50, especially Basal and our subtype 2. Furthermore, in order to compare the performance on pathway activity between different subtypes, the enrichment analysis performed on the mRNA expression of BIC is shown in Supplementary Fig. S5.

Supplementary Figs S6–S11 and Table S8 show the results of sensitivity analysis that how the performance varies with parameters *α* and *β*. Their values are selected from {0.2,0.5,1,5,10}. From these figures, we can observe that the MRGCN is robust with respect to trade-off parameters of loss function, hence, for simplicity, *α* and *β* could be always set to 1. In addition, Supplementary Figs S12 and S13 display subtyping results in different dimensions of the consensus representation. It also can be found that MRGCN is relatively robust with respect to the dimension of consensus representation.

The generalization capability of the proposed method is carried out in 2-fold validation study. The first fold is on BIC and METABRIC datasets. METABRIC has two omics, but BIC has three omics. The overlapped expression between them is only mRNA. Thus, the four clusters of BIC are already obtained via the proposed MRGCN trained on three omics. Then, each sample of METABRIC is classified into the four clusters of BIC using shrunken centroid classifier (Tibshirani *et al.* 2002) via mRNA profiles of METABRIC and the cluster centroids of BIC. Supplementary Fig. 14a and b shows the sample distributions and PAM50+Claudin-low subtype compositions of the identified clusters. Supplementary Fig. 14 (c) shows the In-Group Proportion (Kapp and Tibshirani 2007) score and *P*-value for each cluster of METABRIC. In the second fold, in order to guarantee the training and validation data have the same multi-omics, we randomly set aside 80% of BIC data for training, 20% for validation. The experimental procedures follow the first fold study, and its results are shown in Supplementary Fig. 15. It can be observed from these figures that identified clusters could be reproduced and the proposed method has good generalization capability on independent datasets.

### 3.2 Partial multi-omics datasets

In order to evaluate the performance of methods on partial multi-omics datasets, we simulate some patients loss omics measurements. Specifically, we randomly sampled a fraction *θ* of the patients and removed their mRNA expression. Consequently, for TCGA datasets miRNA and DNA methylation expression are full, and for METABRIC dataset CNV expression is full. The survival analysis and enrichment of clinical labels are still adopted to evaluate the quality of methods. The performance is presented in Table 2 and Fig. 3. Table and figure reveal that MRGCN gives a better performance than MSNE, NEMO, and MCCA with respect to survival and enrichment analysis under all missing rates. These results demonstrates that MRGCN is robustly applied to partial omics missing situation. Generally, cancer subtyping via MRGCN with statistically significant difference in survival profiles and significant clinical enrichment. In addition, MRGCN can effectively tackle the partial omics missing challenge.

**Table 2.** The comparisons of clustering results from different algorithms on partial multi-omics TCGA and METABRIC datasets.^a^

Alg./cancer	AML	BIC	COAD	GBM	KIRC	LIHC	LUSC	OV	SKCM	SARC	METABRIC	Mean	Sig
	*θ *= 0.1												
MCCA	1/**3.9**	1/**3.5**	0/0.5	1/**2.0**	2/**2.2**	1/0.7	1/1.2	1/0.3	1/**2.7**	0/0.8	1/**5.2**	0.9/**2.1**	9/6
MSNE	1/**3.5**	2/**3.9**	0/0.3	1/**2.5**	2/**1.5**	2/**1.5**	1/**2.5**	0/0.8	1/1.0	2/**1.6**	1/**2.0**	1.2/**1.9**	9/8
NEMO	1/**3.1**	2/**4.3**	1/0.1	1/**2.8**	3/**1.5**	3/**2.8**	1/**2.2**	0/0.1	1/0.5	2/0.9	1/**1.7**	1.5/**1.8**	10/7
MRGCN	1/**4.1**	2/**5.9**	1/0.7	2/**3.5**	4/**1.7**	2/0.9	1/**2.5**	0/**1.4**	2/**2.5**	2/**3.4**	1/**2.9**	1.6/**2.7**	10/9
	*θ *= 0.3												
MCCA	1/**2.2**	2/**4.0**	0/0.2	1/0.4	0/**1.5**	1/1.3	0/0.8	1/0.1	0/**1.7**	0/0.9	1/**1.9**	0.6/**1.4**	6/5
MSNE	1/**2.4**	2/**3.6**	0/0.3	1/**2.0**	2/1.3	2/**1.7**	0/**1.8**	1/0.6	0/1.1	1/1.0	0/**1.4**	0.9/**1.6**	7/6
NEMO	1/**2.4**	2/**4.0**	1/0.3	1/**1.6**	3/1.2	3/**3.8**	0/0.7	1/0.3	0/0.2	2/0.9	0/**1.6**	1.3/**1.5**	8/5
MRGCN	1/**2.5**	2/**4.8**	1/0.5	2/**4.6**	3/1.0	2/0.8	1/0.8	1/1.3	2/**3.6**	2/**3.5**	0/**1.8**	1.5/**2.3**	10/6
	*θ *= 0.5												
MCCA	1/**2.8**	1/**4.1**	0/0.3	1/**1.9**	2/**2.7**	1/0.6	0/0.8	0/0.1	2/1.1	1/0.4	1/**1.4**	0.9/**1.5**	8/5
MSNE	1/**2.5**	2/**3.5**	0/0.4	1/**2.1**	2/**1.4**	1/1.0	1/1.2	0/0.3	0/1.0	1/**1.5**	0/**1.5**	0.8/**1.5**	7/6
NEMO	1/**3.1**	2/**4.7**	1/0.1	1/**2.7**	1/1.2	2/**1.9**	1/1.1	0/0.1	0/0.3	2/**2.1**	0/**1.5**	1.0/**1.7**	8/6
MRGCN	1/**3.2**	1/**5.1**	1/0.1	2/**4.1**	2/**1.5**	2/0.4	0/**1.5**	0/0.7	1/0.6	2/**2.7**	0/**1.6**	1.1/**2.0**	8/7
	*θ *= 0.7												
MCCA	1/**2.8**	1/**3.8**	0/0.3	1/**2.5**	2/**2.6**	1/1.3	0/1.3	0/0.1	2/**2.4**	0/1.0	0/**2.0**	0.7/**1.8**	6/6
MSNE	1/**2.5**	2/**3.5**	0/0.4	1/**2.6**	2/**1.8**	1/1.1	1/1.1	0/0.3	0/0.9	1/**1.5**	0/**1.5**	0.8/**1.6**	7/6
NEMO	1/**2.9**	2/**4.5**	1/0.1	1/**3.3**	4/**2.2**	2/**1.9**	0/1.1	0/0.1	0/0.3	2/0.9	0/**1.4**	1.2/**1.7**	7/6
MRGCN	1/**2.4**	1/**5.0**	1/0.3	2/**5.0**	4/**1.8**	2/0.6	0/1.1	0/0.2	1/1.1	2/**2.8**	0/**2.0**	1.3/**2.0**	8/6

a *θ* is the fraction of missing data. In each cell A/B, A is significant clinical parameters detected. B is −log10 *P*-value for survival. 0.05 is the threshold for significance and the bold indicates the significant results. Mean is algorithm average value. Sig is the number of datasets with significant results.

## 4 Discussion and conclusion

Cancer subtyping plays an important role in targeted treatment and precision medicine, and ultimately helps to increase survival chances of cancer patients. Cancer is a phenotypic end-point of event accumulated by multiple levels of biological system from genome to proteome. Multi-omics data integration can improve understanding of underlying biological mechanisms and improves clinical outcome. An effective cancer subtyping framework, namely MRGCN is presented for multi-omics integration and clustering. Different from existing integrative approaches, MRGCN aims at preserving omics expression and similarity relationships simultaneously. Also, MRGCN aims to deal with the tackle of some samples missing values on partial omics. To solve these problems efficiently, the reconstruction GCN and indicator matrix are designed and applied. Based on 10 TCGA and 1 METABRIC multi-omics datasets, computational experimental results indicate that MRGCN can provide better integrative performance. Although two or three levels omics are used in experiments, MRGCN is an open framework and could easily be utilized in more omics scenarios. We believe that MRGCN will ultimately lay the foundations for refined representation and understanding of diseases. Another important future work is to involve protein–protein interaction networks to improve the interpretability of integrative embedding.

## Supplementary Material

btad353_Supplementary_Data
