# Supplementary material for: MRGCN: cancer subtyping with multi-reconstruction graph convolutional network using full and partial multi-omics dataset
Source: Bioinformatics. 2023 May 31;39(6):btad353. doi: 10.1093/bioinformatics/btad353 (PMC10279523; doi:10.1093/bioinformatics/btad353)
Supplement: btad353_Supplementary_Data [file btad353_supplementary_data.pdf]

# Supplementary Materials for “MRGCN: cancer subtyping with multi-reconstruction graph convolutional network using full and partial multi-omics dataset”

Supplementary Table 1: The size of preprocessed datasets.

| | AML | BIC | COAD | GBM | KIRC | LIHC | LUSC | OV | SKCM | SARC | METABRIC |
|---|---|---|---|---|---|---|---|---|---|---|---|
| Size | 170 | 624 | 220 | 274 | 184 | 367 | 341 | 287 | 450 | 257 | 1904 |

Supplementary Table 2: Number of clusters.

| | AML | BIC | COAD | GBM | KIRC | LIHC | LUSC | OV | SKCM | SARC | METABRIC |
|---|---|---|---|---|---|---|---|---|---|---|---|
| K-means | 5 | 4 | 2 | 5 | 2 | 2 | 2 | 2 | 2 | 2 | 5 |
| Spectral | 6 | 3 | 12 | 5 | 3 | 2 | 2 | 4 | 6 | 2 | 7 |
| LRACluster | 7 | 5 | 10 | 12 | 11 | 12 | 12 | 4 | 15 | 13 | 7 |
| CC | 3 | 5 | 2 | 7 | 4 | 2 | 4 | 3 | 4 | 2 | 6 |
| PINS | 4 | 5 | 4 | 2 | 6 | 5 | 2 | 2 | 15 | 3 | 7 |
| MCCA | 12 | 5 | 2 | 11 | 15 | 15 | 12 | 9 | 2 | 15 | 7 |
| iClusterBayes | 5 | 4 | 2 | 2 | 2 | 6 | 5 | 6 | 2 | 2 | 7 |
| SNF | 6 | 5 | 3 | 2 | 4 | 5 | 2 | 3 | 3 | 3 | 7 |
| SNFCC | 4 | 5 | 10 | 9 | 2 | 10 | 2 | 3 | 4 | 3 | 7 |
| MSNE | 5 | 4 | 5 | 2 | 4 | 5 | 2 | 4 | 4 | 3 | 9 |
| NEMO | 5 | 4 | 3 | 4 | 12 | 5 | 2 | 3 | 5 | 3 | 9 |
| MRGCN | 10 | 4 | 7 | 8 | 9 | 10 | 13 | 5 | 5 | 8 | 9 |

Supplementary Table 3: The hyper-parameters of comparison methods.

| | Parameters |
|---|---|
| K-means | maximum iteration∈{50,100,200,300,400,500} |
| Spectral | K∈{5,10,15,20} |
| LRACluster | dimension∈{2,3,…,10}, type="gaussian" |
| CC | clusterAlg = "hc", distance = "pearson", reps = 20, pItem = 0.8, pFeature = 1 |
| PINS | agreementCutoff∈{0.2,0.3,0.4,0.5}, ncore = 1 |
| MCCA | niter∈{15,20,25,30}, nstart∈{10,20,30} |
| iClusterBayes | pp.cutoff∈{0.2,0.4,0.6,0.8} |
| SNF | K∈{10,15,20}, alpha∈{0.3,0.4,…,0.8} |
| SNFCC | K∈{10,15,20}, alpha∈{0.3,0.4,…,0.8}, reps∈{10,20,…,50} |
| MSNE | window size∈{10,20,30}, walk length∈{10,15,20}, number of local neighbors∈{10,15,20} |
| NEMO | num_neighbors=number of samples/number of samples clusters |

Note: The key parameters in each method are tested from the candidate sets. For different cancer dataset, it has different optimal parameter value, hence we use silhouette value to choose the optimal parameter value.

Supplementary Table 4: The dimension of consensus representation $\boldsymbol{H}$.

| | AML | BIC | COAD | GBM | KIRC | LIHC | LUSC | OV | SKCM | SARC | METABRIC |
|---|---|---|---|---|---|---|---|---|---|---|---|
| $d$ | 446 | 708 | 490 | 427 | 632 | 681 | 704 | 492 | 720 | 670 | 1600 |

Supplementary Table 5: The numbers of nodes in each layer on TCGA datasets.

| | | AML | BIC | COAD | GBM | KIRC | LIHC | LUSC | OV | SKCM | SARC |
|---|---|---|---|---|---|---|---|---|---|---|---|
| | $N_I$ | 2000 | 2000 | 2000 | 2000 | 2000 | 2000 | 2000 | 2000 | 2000 | 2000 |
| mRNA | $N_E$ | 1600 | 1600 | 1600 | 1600 | 1600 | 1600 | 1600 | 1600 | 1600 | 1600 |
| | $N_R$ | 446 | 708 | 490 | 427 | 632 | 681 | 704 | 492 | 720 | 670 |
| | $N_I$ | 2000 | 2000 | 2000 | 2000 | 2000 | 2000 | 2000 | 2000 | 2000 | 2000 |
| methylation | $N_E$ | 1600 | 1600 | 1600 | 1600 | 1600 | 1600 | 1600 | 1600 | 1600 | 1600 |
| | $N_R$ | 446 | 708 | 490 | 427 | 632 | 681 | 704 | 492 | 720 | 670 |
| | $N_I$ | 558 | 885 | 613 | 534 | 791 | 852 | 881 | 616 | 901 | 838 |
| miRNA | $N_E$ | 446 | 708 | 490 | 427 | 632 | 681 | 704 | 492 | 720 | 670 |
| | $N_R$ | 446 | 708 | 490 | 427 | 632 | 681 | 704 | 492 | 720 | 670 |

Supplementary Table 6: The numbers of nodes in each layer on METABRIC.

| | | METABRIC |
|---|---|---|
| mRNA | $N_I$ | 2000 |
| | $N_E$ | 1600 |
| | $N_R$ | 1600 |
| CNV | $N_I$ | 2000 |
| | $N_E$ | 1600 |
| | $N_R$ | 1600 |

Note: MRGCN has a 6 layers structure, i.e., Input layer, Encoder layer, Representation layer, Consensus representation layer, Decoder layer, Output layer. The numbers of nodes in each layer are noted as $N_I$, $N_E$, $N_R$, $N_C$, $N_D$, $N_O$, respectively. The guideline to the numbers of nodes in each layer is designed as follows:

(1) Input layer: If the original feature dimension of the given omics data>2000, compute and rank the standard deviations of each feature, and the 2000 top ranked features are selected as input. Then set $N_I$=2000. If original feature dimension of the given omics data≤2000, all features are considered as input. Then $N_I$ is set to the feature dimension.

(2) Encoder layer: If $N_I$=2000, then set $N_E$=1600, (i.e., 0.8*2000 for dimension reduction). If $N_I$ <2000, then set $N_E$=$N_C$.

(3) Representation layer: If the lowest dimension in all omics<2000, set $N_C$=0.8*the lowest dimension in all omics, else set $N_R$=1600.

(4) Consensus representation layer: $N_C$=$N_R$.

(5) Decoder layer: Set $N_D$=$N_E$.

(6) Output layer: Set $N_O$=$N_I$.

Since the networks have symmetric structure, i.e., $N_C$=$N_R$, $N_D$=$N_E$ and $N_O$=$N_I$, thus we only give the $N_I$, $N_E$, $N_R$ for each dataset.

Supplementary Table 7: Significant clinical parameters found by different algorithms on full TCGA and METABRIC datasets.

| | AML | BIC | COAD | GBM | KIRC | LIHC | LUSC | OV | SKCM | SARC | METARBIC |
|---|---|---|---|---|---|---|---|---|---|---|---|
| K-means | A | N, T | S | A, G | M | A, G | 0 | A | A, S | A, G | 0 |
| Spectral | A | N | S | A, G | G, M, S | A, G | 0 | A | T, S | A, G | 0 |
| LRACluster | A | N, S | S | A, G | G, M, T, S | A, G | A | A | A, T, S | A, G | 0 |
| CC | A | M | S | A, G | M, T, S | A, G | A | 0 | A, T, S | A, G | A |
| PINS | A | N | 0 | A | G, M, T | A, G | 0 | 0 | A, S | A, G | A |
| MCCA | A | N | 0 | A | M, T | A, G | A, G | 0 | T, S | A, G | A |
| iClusterBayes | A | N, T | M, S | A | G, M, T, S | A, G | 0 | A | A, S | A, G | A |
| SNF | A | N, T | S | A, G | M, T, S | A, G | A | A | A | A, G | 0 |
| SNFCC | A | N, T, S | A, S | A, G | M, T | A | A | 0 | A, N | A, G | A |
| MSNE | A | N, T | S | A | M, T | A, G, M | A | 0 | A, S | A, G | A |
| NEMO | A | A, N | 0 | A | G, M, T, S | A, G, M, N, | 0 | 0 | A, T, S | A, G | A |
| MRGCN | A | A, N, T, S | S | A, G | G, M, T, S | A, G, | A | A | A, T, S | A, G | A |

Note: A denotes age. G denotes gender. T denotes progression of the tumor. N denotes cancer in lymph nodes. M denotes metastases. S denotes total progression. 0 is no significant clinical parameter found.

Supplementary Table 8: Adjusted Rand Index (ARI) and Normalized Mutual Information (NMI) between the cluster assignments of BIC dataset obtained by using different $\alpha$ and $\beta$ values (compared with $\alpha=1$, $\beta=1$).

| $\beta / \alpha$ | 0.2 | 0.5 | 1 | 5 | 10 |
|---|---|---|---|---|---|
| | | | ARI | | |
| 0.2 | 0.8890 | 0.9460 | 0.9470 | 0.6767 | 0.6232 |
| 0.5 | 0.7062 | 0.9286 | 0.9399 | 0.6514 | 0.6416 |
| 1 | 0.7288 | 0.9561 | 1 | 0.6729 | 0.6678 |
| 5 | 0.6966 | 0.9419 | 0.9566 | 0.7460 | 0.5835 |
| 10 | 0.8683 | 0.9364 | 0.9562 | 0.8591 | 0.6491 |
| | | | NMI | | |
| 0.2 | 0.8275 | 0.9103 | 0.9252 | 0.7387 | 0.7701 |
| 0.5 | 0.7093 | 0.9286 | 0.9128 | 0.8000 | 0.7920 |
| 1 | 0.7320 | 0.9341 | 1 | 0.7850 | 0.7681 |
| 5 | 0.7069 | 0.9012 | 0.9338 | 0.8348 | 0.7984 |
| 10 | 0.8165 | 0.8946 | 0.9292 | 0.8976 | 0.7725 |

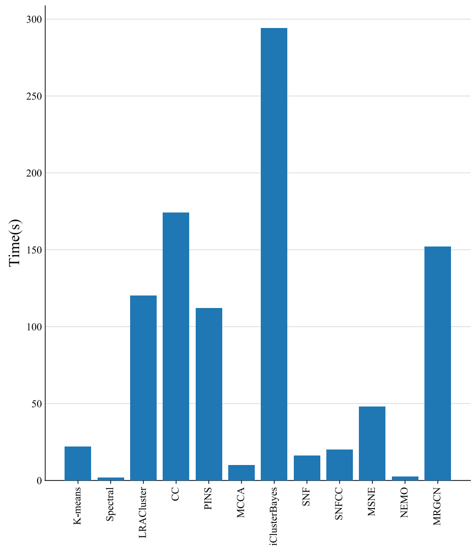


Supplementary Fig 1. Time consumption of different methods on BIC dataset.

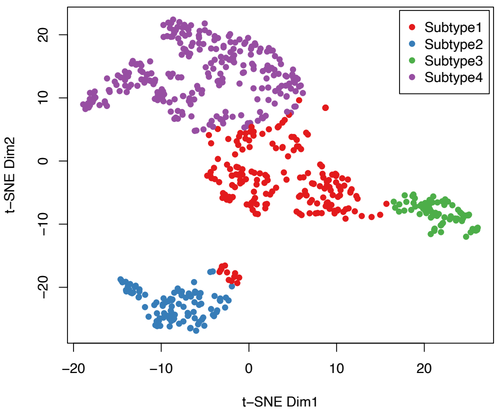


(a) BIC via t-SNE

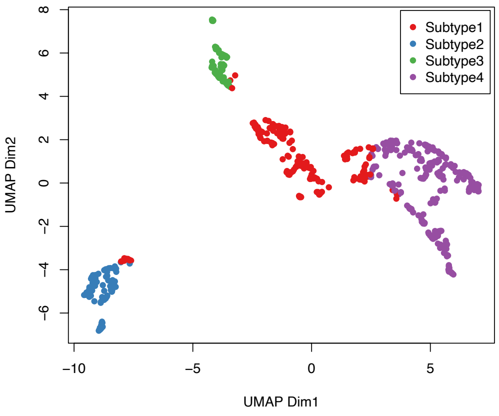


(b) BIC via UMAP

Supplementary Fig 2. The visualization of MRGCN on BIC dataset.

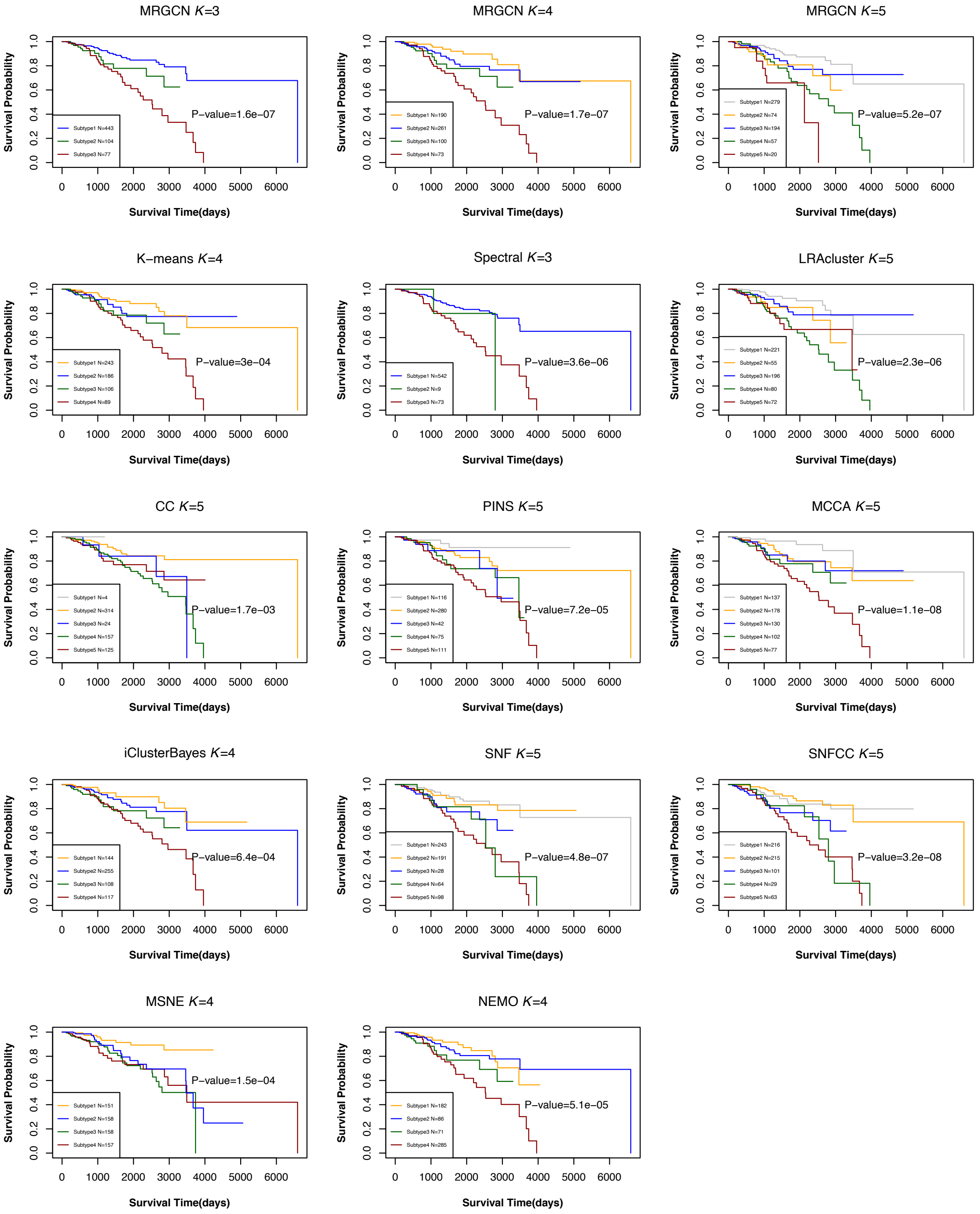


Supplementary Fig 3. Kaplan Meier survival curves of BIC subtypes. N is the number of samples in each cluster and K is the number of clusters. In experiments the proposed MRGCN divides the BIC data into 4 clusters, but in order to directly compare with other state-of-the-art methods, the number of clusters in MRGCN is set range from 3 to 5, we give the survival curves of MRGCN based on the number in clustering 3, 4 and 5 respectively.

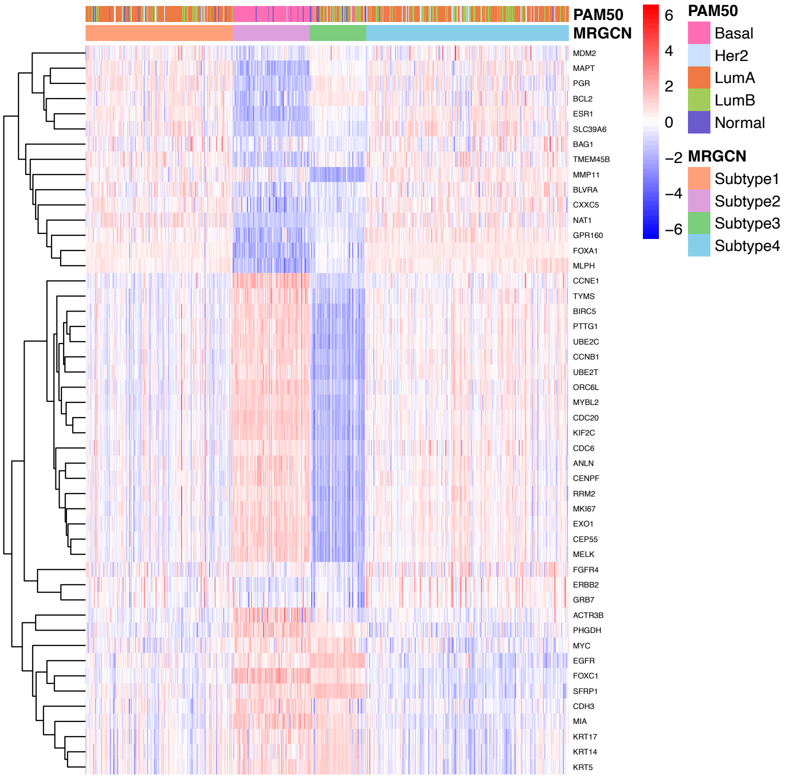


Supplementary Fig 4. The heatmap for BIC dataset. The top bar indicates the PAM50 annotation of the samples together with the subtyping results of MRGCN. The columns correspond to patients, and the rows correspond to the mRNA related to PAM50.

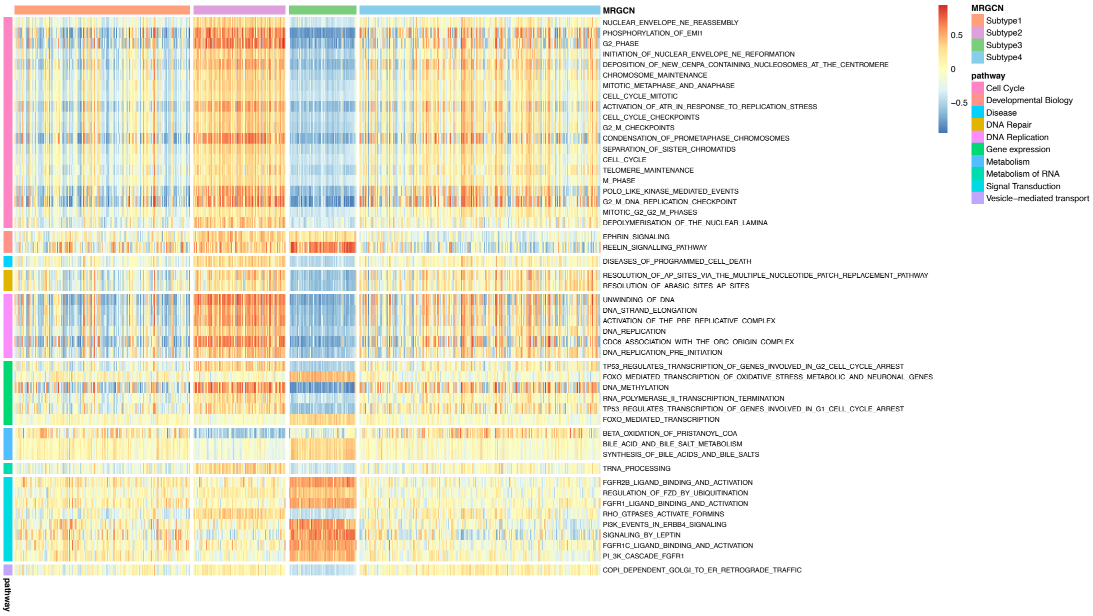


Supplementary Fig 5. Top 50 enriched pathways in four subtypes of BIC. Gene set variation analysis provides an estimate of pathway activity by transforming an input gene-by-sample expression data matrix into a corresponding gene-set-by-sample expression data matrix. In order to compare the performance on pathway activity between different subtypes obtained by MRGCN, the enrichment analysis was performed on the mRNA expression matrix of BIC (related to all 20501 genes) and the "c2.cp.reactome.v7.4.symbols.gmt" gene set in MSigDB, using the R package "GSVA". The R package "limma" is used to analyze the difference of pathways.

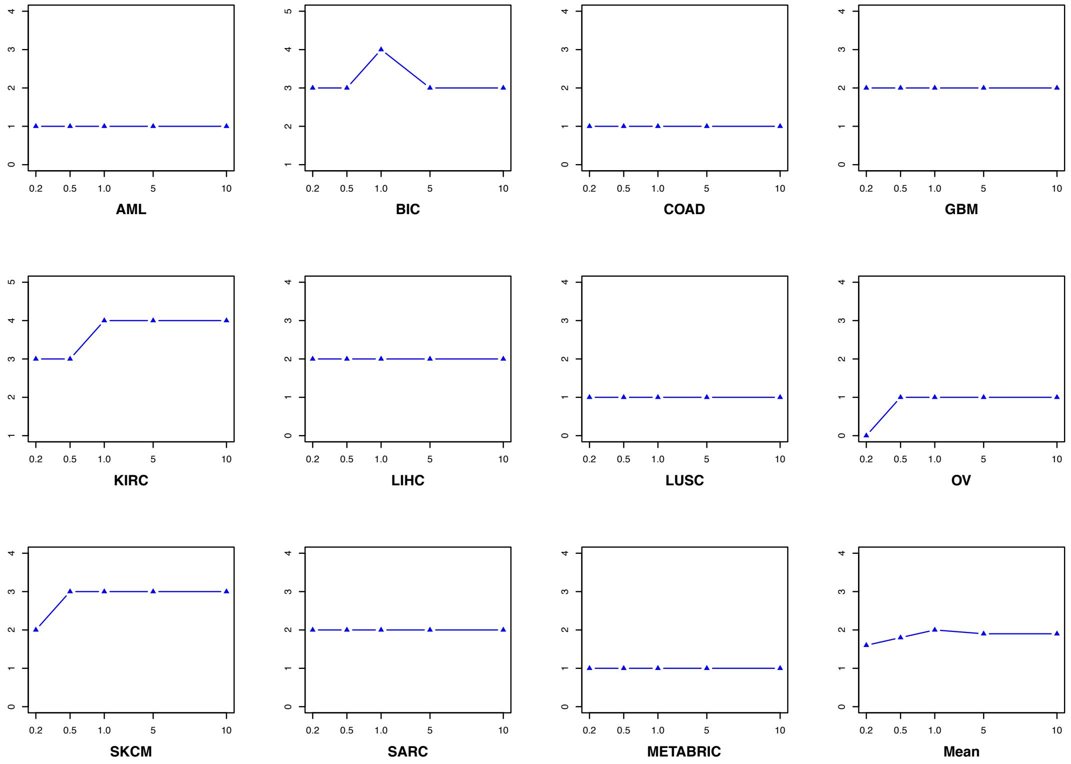


Supplementary Fig 6. Sensitivity analysis of clinical parameters for MRGCN with respect to $\alpha$ . For each dataset, MRGCN is run using different values for $\alpha$ and keeping $\beta$ =1, and report the number of significantly enriched clinical parameters when comparing the obtained clusters. X-axis represents the value of $\alpha$ . Mean stands for the average values over all datasets.

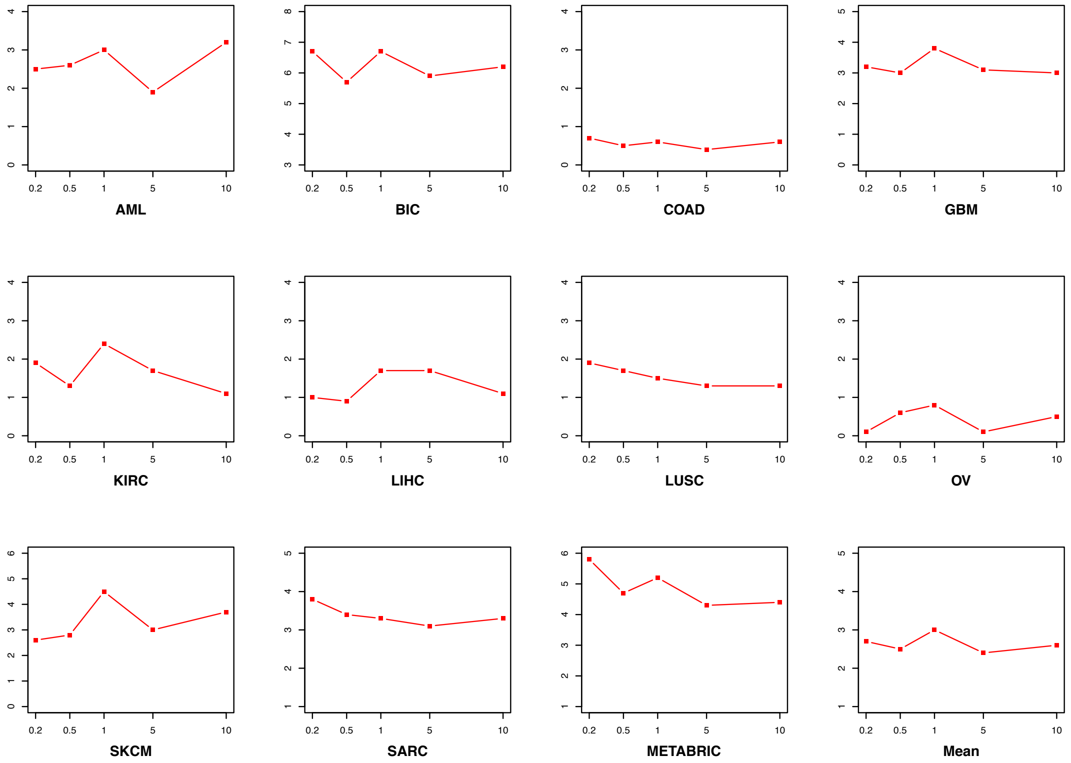


Supplementary Fig 7. Sensitivity analysis of survival for MRGCN with respect to $\alpha$ . For each dataset, MRGCN is run using different values for $\alpha$ and keeping $\beta$ =1, and report the results of logrank test for comparing survival between the obtained clusters. Y-axis is -log10 of the logrank test P-value. X-axis represents the value of $\alpha$ . Mean stands for the average values over all datasets.

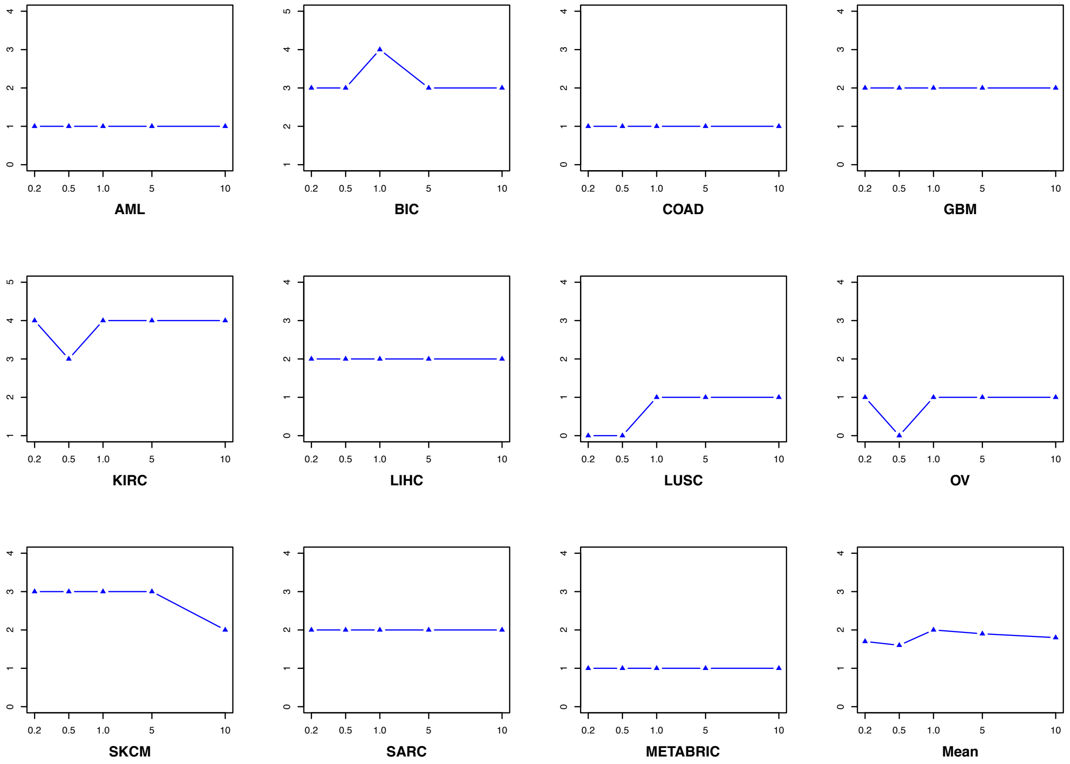


Supplementary Fig 8. Sensitivity analysis of clinical parameters for MRGCN with respect to $\beta$ . For each dataset, MRGCN is run using different values for $\beta$ and keeping $\alpha$ =1, and report the number of significantly enriched clinical parameters when comparing the obtained clusters. X-axis represents the value of $\beta$ . Mean stands for the average values over all datasets.

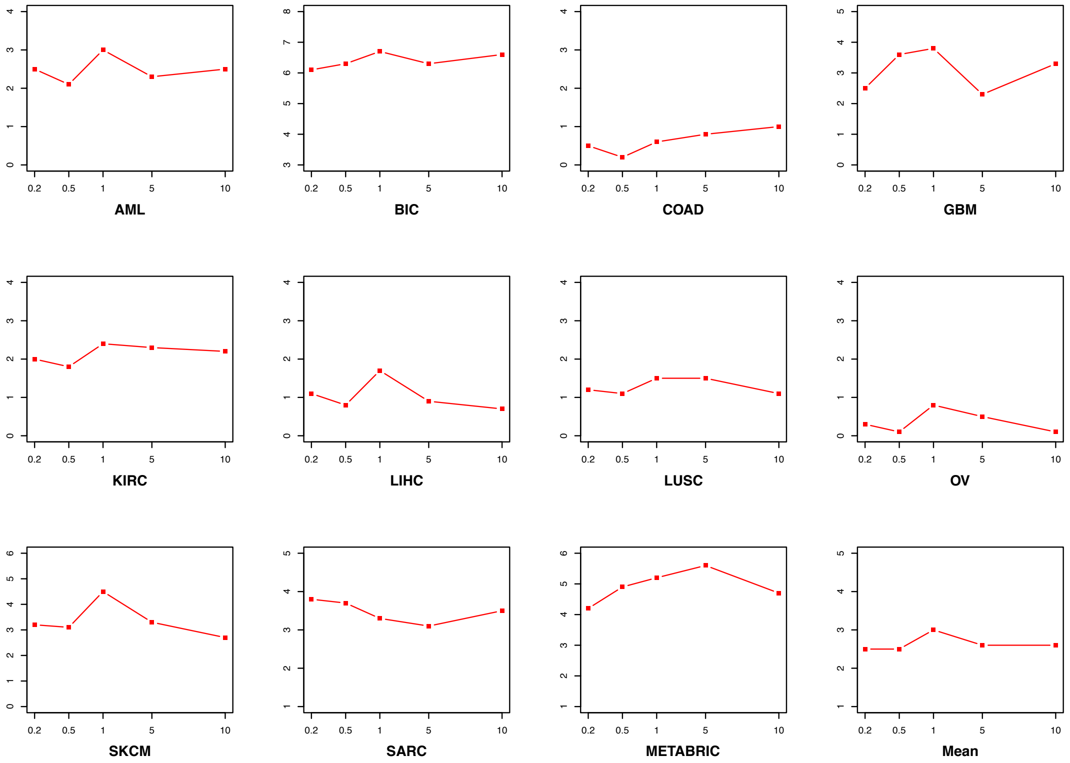


Supplementary Fig 9. Sensitivity analysis of survival for MRGCN with respect to $\beta$ . For each dataset, MRGCN is run using different values for $\beta$ and keeping $\alpha$ =1, and report the results of logrank test for comparing survival between the obtained clusters. Y-axis is -log10 of the logrank test P-value. X-axis represents the value of $\beta$ . Mean stands for the average values over all datasets.

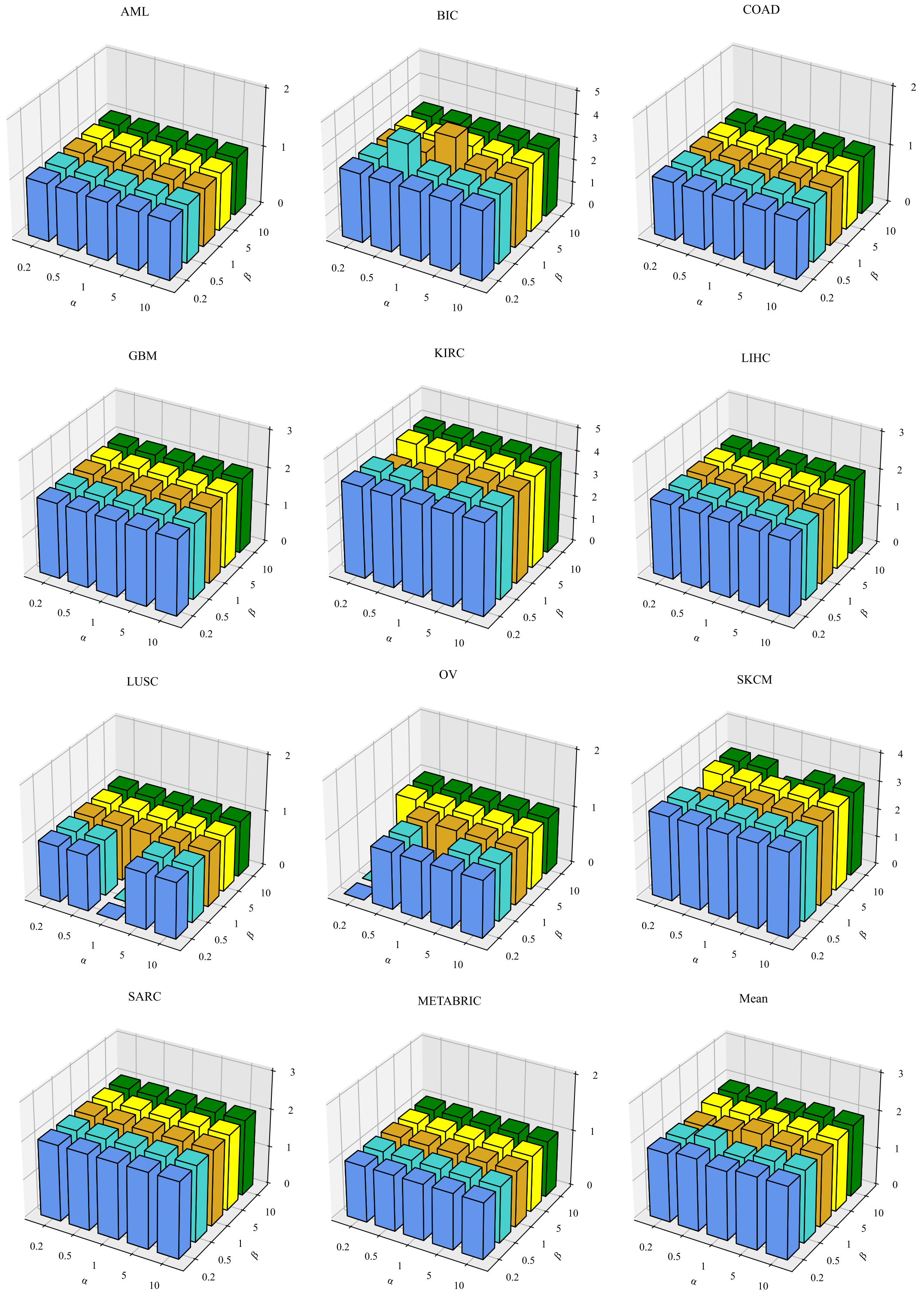


Supplementary Fig 10. Sensitivity analysis of clinical parameters for MRGCN with respect to $\alpha$ and $\beta$ .

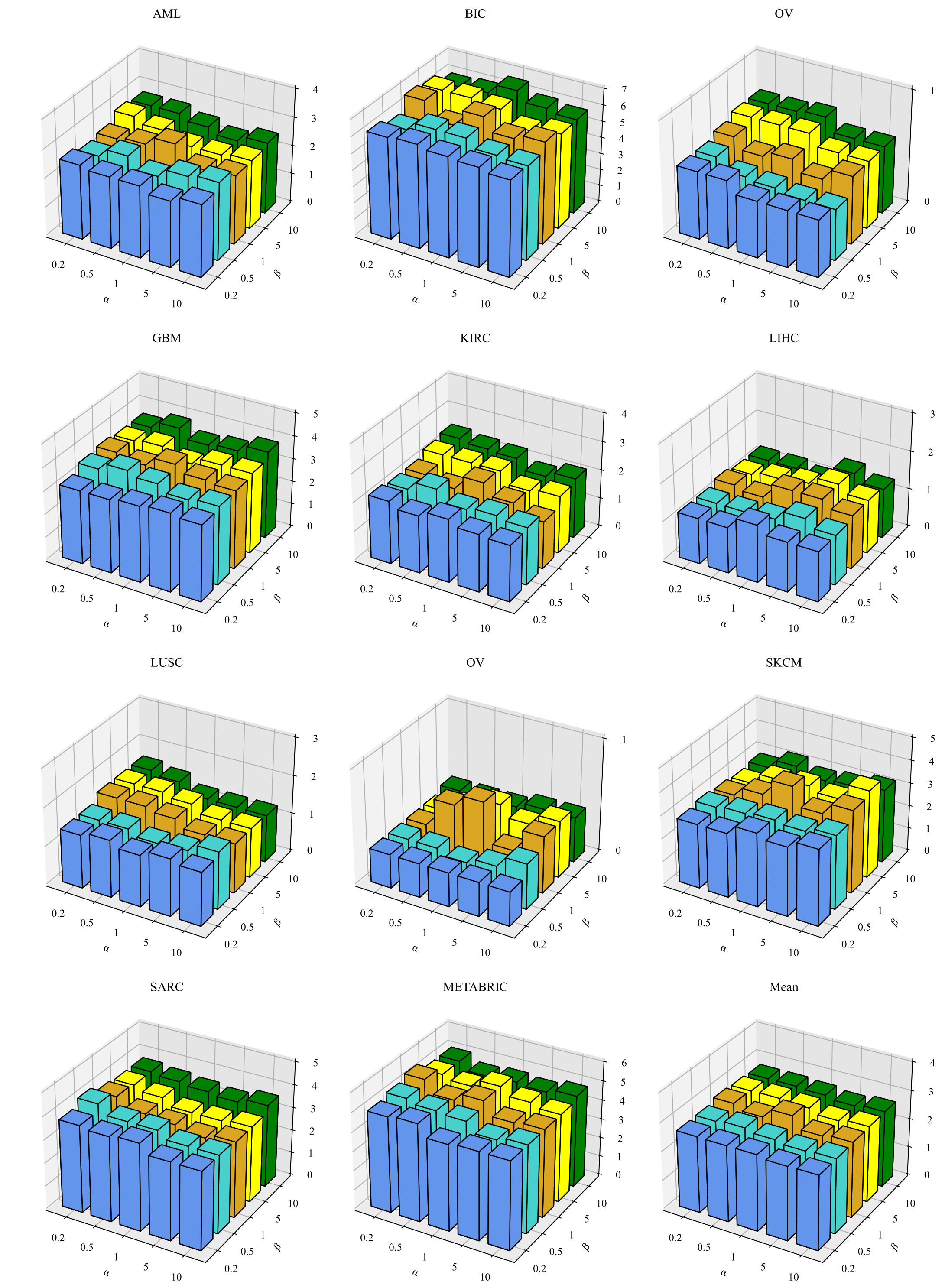


Supplementary Fig 11. Sensitivity analysis of survival for MRGCN with respect to $\alpha$ and $\beta$ .

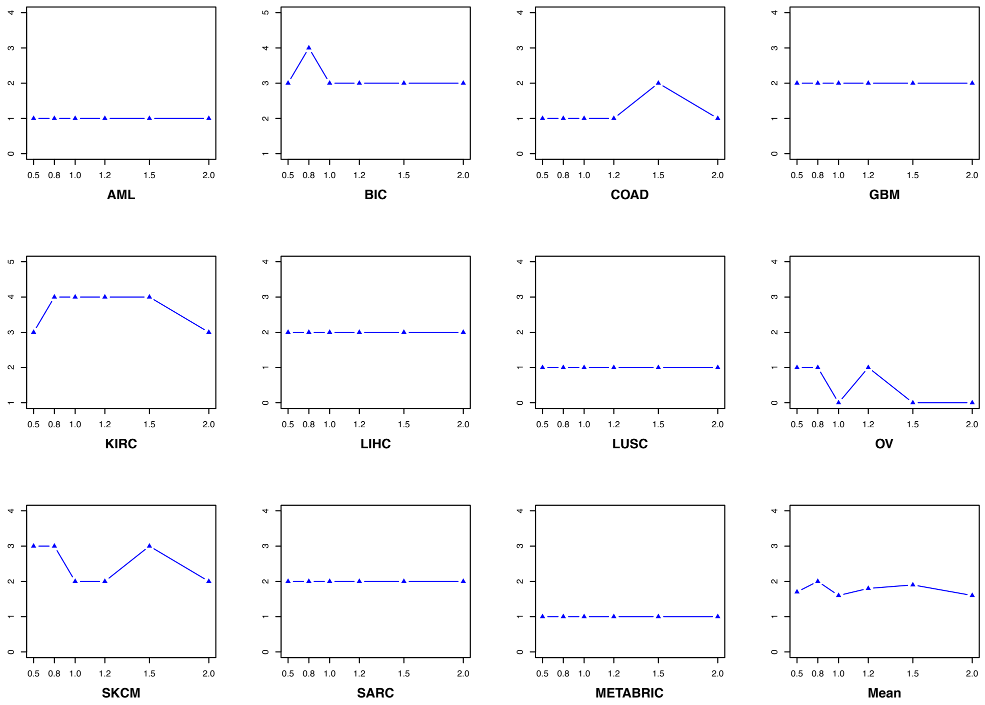


Supplementary Fig 12. Sensitivity analysis of clinical parameters for MRGCN with respect to the dimension of consensus representation. For each dataset, MRGCN is run using different values for dimension of consensus representation, and report the number of significantly enriched clinical parameters when comparing the obtained clusters. X-axis represents compression ratios of the lowest dimension in all omics. Mean stands for the average values over all datasets.

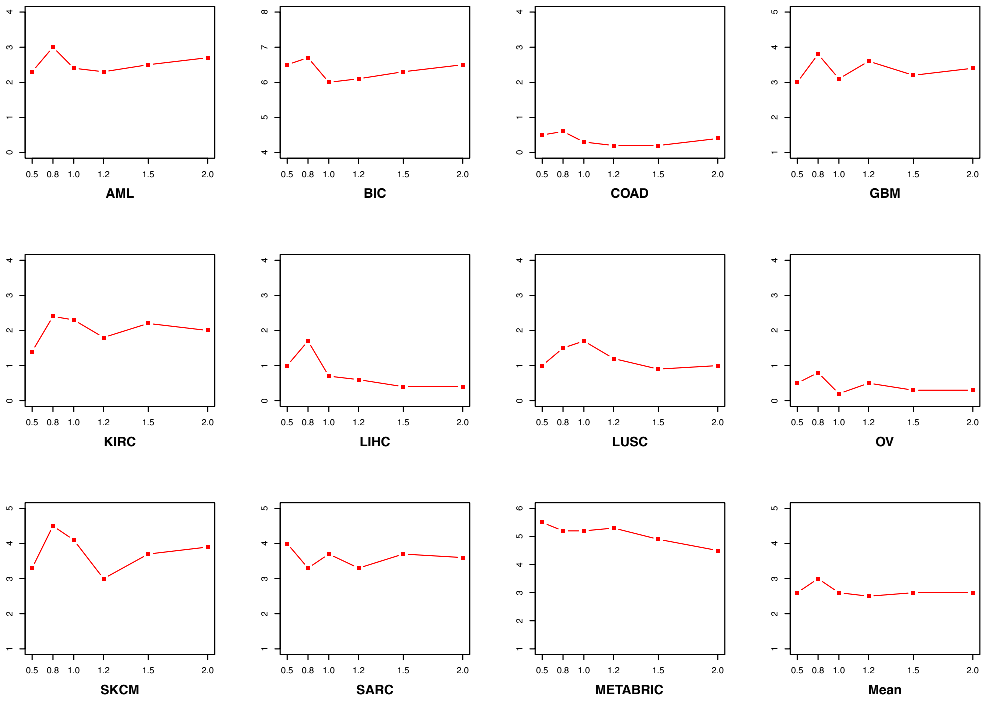


Supplementary Fig 13. Sensitivity analysis of survival for MRGCN with respect to the dimension of consensus representation. For each dataset, MRGCN is run using different values for dimension of consensus representation, and report the results of logrank test for comparing survival between the obtained clusters. Y-axis is -log10 of the logrank test P-value. X-axis represents compression ratios of the lowest dimension in all omics. Mean stands for the average values over all datasets.

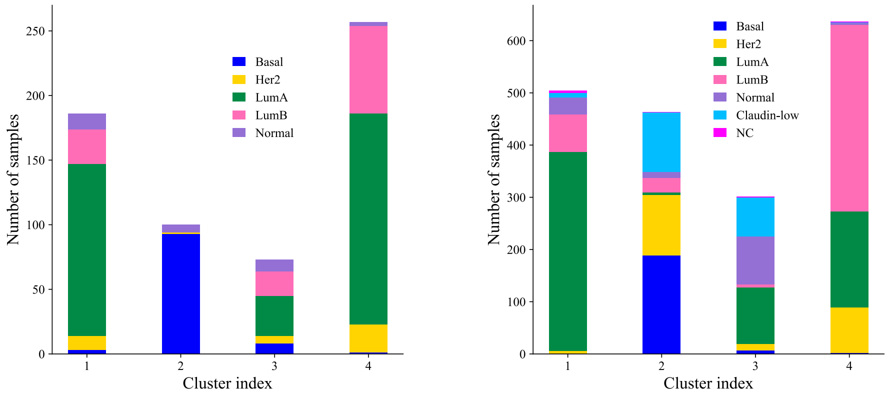


(a) (b)

| Cluster ID | 1 | 2 | 3 | 4 |
|---|---|---|---|---|
| IGP | 0.71 | 0.90 | 0.86 | 0.76 |
| P-value | 0 | 0 | 0 | 0 |

(c)

Supplementary Fig 14. Validation results on the BIC and METABRIC datasets. (a-b) The clusters detected in the BIC (top left) and METABRIC datasets (top right) compared with PAM50+Claudin-low subtype compositions. (c) IGP scores and P-values of clusters in METABRIC.

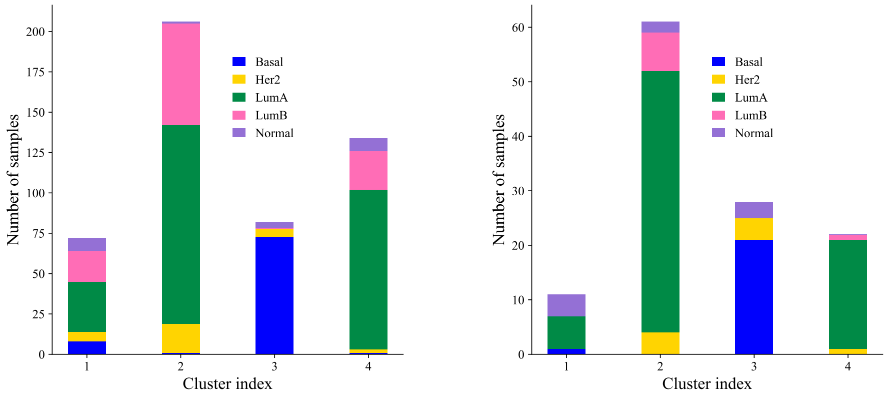


(a) (b)

| Cluster ID | 1 | 2 | 3 | 4 |
|---|---|---|---|---|
| IGP | 0.82 | 0.97 | 0.96 | 0.95 |
| P-value | 0 | 0 | 0 | 0 |

(c)

Supplementary Fig 15. Validation results on the BIC training and validation datasets. (a-b) The clusters detected in the BIC training (top left) and BIC validation datasets (top right) compared with PAM50+Claudin-low subtype compositions. (c) IGP scores and P-values of clusters in BIC validation.
